# Formulation and Comprehensive Evaluation of Biohybrid Hydrogel Membranes Containing Doxycycline or Silver Nanoparticles

**DOI:** 10.3390/pharmaceutics15122696

**Published:** 2023-11-28

**Authors:** Diana Stan, Lavinia Liliana Ruta, Lorena-Andreea Bocancia-Mateescu, Andreea-Cristina Mirica, Dana Stan, Marin Micutz, Oana Brincoveanu, Ana-Maria Enciu, Elena Codrici, Ionela Daniela Popescu, Maria Linda Popa, Flaviana Rotaru, Cristiana Tanase

**Affiliations:** 1 DDS Diagnostic, Segovia 1 Str., 031427 Bucharest, Romania; lavinia.ruta@chimie.unibuc.ro (L.L.R.); research@ddsdiagnostic.com (L.-A.B.-M.); research.imuno@ddsdiagnostic.com (A.-C.M.); dana_stan@ddsdiagnostic.com (D.S.); 2 Doctoral School of Medicine, Titu Maiorescu University, 040441 Bucharest, Romania; 3 Department of Inorganic, Organic Chemistry, Biochemistry and Catalysis, Faculty of Chemistry, University of Bucharest, 90–92 Panduri Str., 050663 Bucharest, Romania; 4 Department of Analytical and Physical Chemistry, University of Bucharest, 4-12 Regina Elisabeta Blvd., 030018 Bucharest, Romania; micutz@gw-chimie.math.unibuc.ro; 5 National Institute for R&D in Microtechnology, 077190 Bucharest, Romania; oana.brincoveanu@imt.ro; 6 Research Institute, The University of Bucharest, 060102 Bucharest, Romania; 7 Victor Babes National Institute of Pathology, 050096 Bucharest, Romania; ana.enciu@ivb.ro (A.-M.E.); elena.codrici@ivb.ro (E.C.); daniela.popescu@ivb.ro (I.D.P.); bioch@vbabes.ro (C.T.); 8 Department of Cell Biology and Histology, Carol Davila University of Medicine and Pharmacy, 050474 Bucharest, Romania; maria_lindabv@yahoo.com; 9 Polytechnic University of Bucharest, Splaiul Independenței 54, 030167 Bucharest, Romania; flaviana.rotaru@gmail.com; 10 Rohealth—Health and Bioeconomy Cluster, Calea Griviţei 6-8, 010731 Bucharest, Romania; 11 Frontier Management Consulting, Calea Griviţei6-8, 010731 Bucharest, Romania; 12 Department of Cell Biology and Clinical Biochemistry, Titu Maiorescu University, 031593 Bucharest, Romania

**Keywords:** hydrogel, alginate, PVA, doxycycline, silver nanoparticles, collagen, hyaluronic acid, dressing

## Abstract

Complicated wounds often require specialized medical treatments, and hydrogels have emerged as a popular choice for wound dressings in such cases due to their unique properties and the ability to incorporate and release therapeutic agents. Our focus was to develop and characterize a new optimized formula for biohybrid hydrogel membranes, which combine natural and synthetic polymers, bioactive natural compounds, like collagen and hyaluronic acid, and pharmacologically active substances (doxycycline or npAg). Dynamic (oscillatory) rheometry confirmed the strong gel-like properties of the obtained hydrogel membranes. Samples containing low-dose DOXY showed a swelling index of 285.68 ± 6.99%, a degradation rate of 71.6 ± 0.91% at 20 h, and achieved a cumulative drug release of approximately 90% at pH 7.4 and 80% at pH 8.3 within 12 h. The addition of npAg influenced the physical properties of the hydrogel membranes. Furthermore, the samples containing DOXY demonstrated exceptional antimicrobial efficacy against seven selected bacterial strains commonly associated with wound infections and complications. Biocompatibility assessments revealed that the samples exhibited over 80% cell viability. However, the addition of smaller-sized nanoparticles led to decreased cellular viability. The obtained biohybrid hydrogel membranes show favorable properties that render them suitable for application as wound dressings.

## 1. Introduction

Wound healing is a complex process that repairs damaged tissues, with acute wounds healing predictably, while chronic wounds require specialized and prolonged treatment [1]. While traditional dressings offer some protection, modern dressings actively promote optimal healing for complex wounds, revolutionizing the wound care [1,2]. The hydrogel market is anticipated to experience a growth rate of 6.5%, reaching USD 22.8 billion by 2032, driven by increased demand for advanced wound-healing products due to factors like an aging population and a rise in chronic wounds. Hydrogels, known for moisture retention and easy application, are increasingly preferred in advanced wound care. The market is segmented by raw material (synthetic, natural, hybrid), with synthetic holding 53.4% market share in 2022. Key players include 3M, Johnson & Johnson, Cardinal Health, Cooper Vision, and Smith & Nephew [3,4]. Hydrogels’ distinct properties render them ideal for wound care, promoting wound protection, cell migration, proliferation, and tissue regeneration. Hydrogel membranes can be customized for various wound types, sizes, and depths, benefiting both clinicians and patients. One of the key advantages of hydrogels lies in their ability to incorporate and release a wide range of bioactive compounds, ensuring their optimal delivery at the wound site. These compounds can include growth factors [5], antimicrobial agents [6,7], anti-inflammatory drugs [8], plant extracts [9], and even stem cells [10]. Notably, hydrogel membranes with bioactive compounds like collagen and hyaluronic acid hold promise for wound healing as they stimulate cell growth, angiogenesis, and tissue regeneration, mimicking the natural extracellular matrix [11].

Doxycycline (DOXY), a member of the tetracycline class of antibiotics, exhibits broad-spectrum activity against both Gram-positive and Gram-negative bacteria by disrupting bacterial protein synthesis through binding to the bacterial ribosome [12]. Although DOXY has antimicrobial activity, the literature findings have highlighted several other biological effects independent of this action. Tetracyclines are believed to possess anti-inflammatory, immunomodulatory, and antioxidant properties, influencing cell proliferation and migration [13,14]. Among these non-antimicrobial effects, the inhibition of MMP is particularly representative of DOXY’s action. The use of DOXY as a MMP inhibitor via topical administration has been reported at doses ranging from 200 to 375 μg [15]. Researchers are exploring the biological effects of tetracyclines that can be achieved at lower, subantimicrobial doses (20–50 mg) compared to the usual antimicrobial dose (100–300 mg) and also reported that topical application of doses up to 10,000 mg/mL did not cause adverse reactions while maintaining the biological effects [16,17,18]. Modulating protease activity is crucial for addressing conditions with increased inflammation and excessive proteolytic activity, like chronic wounds. In chronic wounds, as in other chronic inflammatory conditions, the elevated levels of pro-inflammatory cytokines (such as tumor necrosis factor [TNF-α] and interleukin [IL-1β]) and matrix metalloproteinases (MMPs) impede the natural healing process. Both animal models and clinical research on ulcerative diseases have indicated that DOXY, an affordable and FDA-approved antibiotic, shows promise in inhibiting MMPs and TNF-α-converting enzyme (TACE), which belongs to the MMP superfamily [19].

In recent years, researchers have been actively exploring alternatives to traditional antibiotic treatments, and one promising option is the use of metallic nanoparticles, such as silver, gold, copper, or titanium nanoparticles [20,21]. The antibacterial properties of silver nanoparticles (npAg) are attributed to the disruptions of the bacterial cell wall (through electrostatic effects and accumulation between lipid layers), the generation of reactive oxygen species (ROS) due to nanoparticle internalization, and the release of positive ions [22,23]. The bactericidal effect is dependent on the size of the nanoparticles (e.g., nanoparticles below 30 nm exhibit increased efficiency in generating ROS) or their shape (e.g., triangular shape more efficient against *E. coli*) [24,25,26,27]. The npAg can be utilized in biomedical material mixtures in low concentrations due to their specific high surface area [28]. Recent research has demonstrated the antimicrobial activity of npAg against a broad spectrum of microorganisms, inhibiting over 650 pathogens, including bacteria, fungi, parasites, and viruses [29]. However, it is crucial to address concerns regarding environmental contamination, cytotoxicity, and potential side effects that may arise from prolonged exposure to npAg. The properties and concentration of npAg can influence their bioactivity, with smaller particles and higher concentrations being more likely to induce adverse effects [30,31,32]. Additionally, the release of silver ions from npAg may contribute to cellular toxicity, impacting both healthy and damaged tissues [33]. Prolonged exposure to npAg within the wound environment may give rise to inflammatory reactions [34] or could result in the accumulation of silver in tissues, potentially causing systemic effects [35,36,37]. To mitigate potential side effects and enhance the safety profile, researchers are exploring various strategies. These include surface modifications [38,39,40], optimization of properties and concentration of npAg [41,42], or incorporation in controlled-release systems [43,44] to minimize cytotoxicity while retaining their therapeutic properties. Currently, there are no internationally agreed investigation standards for nanomaterials [45]. International Standards Organization (ISO) [46], the International Life Science Institute [47], the Institute of Food Science and Technology (IFST) [48], and the European Food Safety Authority (EFSA) [49] are some of the organizations that aim to regulate safety and health issues of nanomaterials.

Both antibiotics and metal nanoparticles have been integrated into a variety of scaffolds, including gels, nanofibers, nanoparticles, foams, and creams, for use as wound dressings. The type of polymer(s) and the crosslinking method stand as pivotal factors governing not only the indication and effectiveness of the end product but also its cost, accessibility, and versatility [50,51]. Erring et al. found that a nanosilver foam dressing was more efficient for re-epithelialization, healing, ease of application, and tolerance when compared to silver nanoparticle gel dressing [52]. Eskitoros-Togay et al. developed core/shell nanofibers containing DOXY for wound treatment, and the results showed good biocompatibility and enhanced drug release; however, the antimicrobial activity was not studied [53]. Abdallah et al. developed a three-dimensional biodegradable electrospun nanofiber scaffold loaded with amoxicillin for wound dressing, drug delivery, and other tissue engineering applications that showed antibacterial activity against *E. coli*, *S. aureus,* and *S. pyogenes* [54]. Several injectable hydrogels containing DOXY and other antibiotics have been formulated for osteoarthritis [55], chronic periodontitis [56], or wound [57,58] treatment. The literature also reports the development of wound dressings that integrate both nanoparticles and antibiotics. Ndlovu et al. developed an alginate-based dissolvable wound dressing incorporating npAg, ampicillin, and ciprofloxacin that exhibited suitable physicochemical properties and demonstrated antibacterial activity [59]. DOXY has mainly been incorporated into nanofibrous scaffolds for wound care applications. While these scaffolds present many advantages, the usage of expensive equipment and an elaborate production process may raise the cost of the final product and limit its availability. Also, the way the treatment is administered can have an impact on the patient’s adherence to the treatment (e.g., injections versus topical). To the best of our knowledge, the production of a hydrogel membrane using the proposed formulations and fabrication process employed in our study has not been previously reported in the existing literature.

In our research, we aim to develop a novel biohybrid hydrogel membrane for wound management that incorporates antimicrobial agents and is designed to be user-friendly, comfortable for patients, and economically viable for production. Our primary objective is to validate the effectiveness of the chosen crosslinking method in preserving the antimicrobial properties of DOXY. Moreover, we have developed formulations containing npAg. A thorough investigation into how the addition of DOXY or npAg influences the physicochemical properties and biocompatibility of the biohybrid hydrogel membrane was also conducted, along with the DOXY release study.

## 2. Materials and Methods

### 2.1. Chemicals and Reagents

All chemical reagents were analytical grade. Polyvinyl alcohol (PVA, Mw = 89,000–98,000, 99% hydrolyzed, MKCP0171, USA), albumin from bovine serum (BSA, fraction V, minimum 96% electrophoresis, nitrogen content 16.2%, 028K0759, USA), calcium chloride (CaCl_2_, pure reagent > 99%, SLBV6838, USA), doxycycline hyclate (DOXY, 0000187261, USA), silver nanoparticles (npAg, citrate stabilized, 20 nm MKCR4722 and 100 nm MKCP3854, USA), glycerol (L125H, Germany), and PEG 200 (BCCB3990, Germany) were purchased from Sigma-Aldrich. Hyaluronic acid (HA, 26337, USA) was purchased from MedChemExpress, and collagen (COL, 354231) from Corning, Europe. Sodium alginate (SA, 19L234104, Belgium) was purchased from VWR Chemicals. CellTiter 96 Aqueous One Solution Cell Proliferation Assay kit was acquired from Promega, Madison, WI, USA. Human foreskin fibroblasts (Hs27, ATCC, CRL-1634, Manassas, VA, USA) were used for cellular studies.

### 2.2. Bacterial Strains and Culture Media

*Staphylococcus epidermidis* (ATCC 12228), *Staphylococcus aureus* (ATCC 700699), *Enterococcus faecalis* (ATCC 19433), *Streptococcus pyogenes* (ATCC 19615), *Pseudomonas aeruginosa* (ATCC 10145), *Acinetobacter baumannii* (ATCC 19606), and *Klebsiella pneumoniae* (ATCC BAA-1705) strains were purchased from MediMark, Europe. Culture media were procured from Avena, Romania, and standardized doxycycline discs (30 μg, 64443624) from Bio-Rad, France.

### 2.3. Equipment

Micrometer (Mytutyo, Japan), pH meter (Thermo Fisher Orion Star A211, Vantaa, Finland), UH5300 Hitachi spectrophotometer (Tokyo, Japan), Anthos Zentyth 3100 spectrophotometer (Anthos, GMBH, Austria), iShak TS4 NXT Microplate Incubator Shaker (GC Life Science, Jaipur, India), Cell-IQ™ Series 5.8 cu.ft. High Heat Sterilization CO_2_ Incubator model MCO-170AICUVDL-PA (PHC Europe, B.V., Nijverheidsweg, The Netherlands), rheometer MFR 2100 (GBC, VIC, Australia), circulating water bath (Lauda Brinkman Ecoline E100, Suite C Marlton, NJ08053, USA), Nova NanoSEM 630 Scanning Electron Microscope (FEI Company, Hillsboro, OR, USA).

### 2.4. Biohybrid Hydrogel Membrane Production

**Figure 1:**
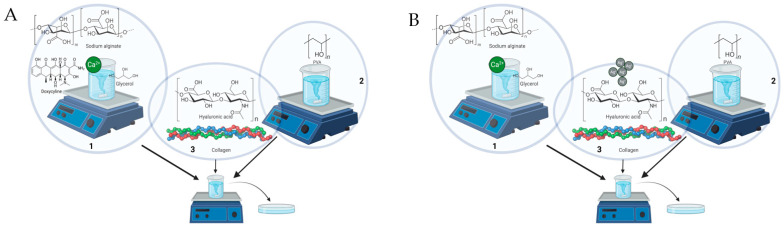
Production process of the hydrogel membranes. (**A**) Doxycycline hydrogel. (**B**) Silver nanoparticles hydrogel. (1). Preparation of alginate suspensions. (2). Preparation of PVA suspension. (3). Adding the final bioactive compounds.

The biohybrid hydrogel membranes (HM) were prepared following the two-step crosslinking process previously described by Stan et al. [60], as depicted in Figure 1 Briefly, to create the hydrogel membranes; PVA (Sigma-Aldrich, USA) was dissolved in distilled water. Separately, SA (VWR Chemicals, Belgium) and CaCl_2_ (Sigma-Aldrich, USA) were dispersed in glycerol (Sigma-Aldrich, Germany), and then the suspension was added to warm distilled water containing doxycycline hyclate (Sigma-Aldrich, USA) in the concentrations mentioned in Table 1. Next, the two polymer solutions were mixed until a homogeneous mixture was obtained, to which bioactive compounds, namely, HA (MedChemExpress, USA) and COL (Corning, Europe), were added. For the production of npAg-containing hydrogel membranes, the appropriate amount of npAg (Sigma-Aldrich, USA) was added to the polymeric and bioactive compound suspension (Table 1). The npAg were solved in PEG 200 (Sigma-Aldrich, Germany) to achieve a final 0.75 mg/mL concentration. The blank hydrogel membranes (HM-C) and pristine hydrogel membranes (HM-Cp) were produced using the same protocol, excluding the incorporation of bioactive components. The mixture was transferred to 5 cm diameter Petri dishes, dried at 28 °C for 72 h, frozen at −20 °C for 20 h, and subjected to three 2-h freezing–thawing cycles for proper crosslinking, following a method adapted from Peppas and Stauffer [61]. The samples were kept in the dark during the production process. To ensure sterility, the samples were exposed to UV light for 15 min. The resulting biohybrid hydrogel membranes were refrigerated in tightly sealed containers until further testing. Table 1 presents the formulations for all the hydrogel membranes, and Figure 1 depicts the production process.

### 2.5. Macroscopic Evaluation and pH

The thickness of the obtained hydrogel membranes was measured using a digital micrometer (Mytutyo, Japan). A total of five points were measured on each sample, and the obtained results are presented as the average value ± standard deviation (S.D.). For the weight variation test, each hydrogel membrane was individually weighed. For pH determination the samples were added to 10 mL of PBS (pH 7.4) and vortexed. The solution was left for 2 h so that all the hydrogel particles were deposited. Later, 5 mL of the solution was taken and analyzed using a digital pH meter (Thermo Fisher Orion Star A211, Vantaa, Finland). All samples were analyzed in triplicate, and the results are presented as mean ± standard deviation (S.D.).

### 2.6. Swelling Index

The swelling index (SI) of the hydrogel membranes was assessed in vitro using the gravimetric method. The simulated wound fluid (SWF, pH 8.4) was prepared following the method described in Arafa’s work [62]. The hydrogel membranes (1.5 cm diameter) were dried for 24–48 h at 55 °C until a constant weight was achieved (Wi). Subsequently, the samples were immersed in excess of SWF (37 °C, 50 rpm) and were weighed (Wf) at predetermined time intervals (5, 30 min, 1, 2, 3, 6, and 24 h). To ensure accurate measurements, excess liquid on the swollen samples was removed using filter paper before weighing, and the consumed liquid was periodically replaced to maintain complete immersion of the samples throughout the test. The SI rate was calculated using the following formula [63]:

SI (%) = (Wf − Wi)/Wi × 100(1)

### 2.7. Water Vapor Uptake

The hydrogel membranes were weighed before (Wi) and after (Wf) being placed in a chamber with a relative humidity of 75% for 72 h. The water vapor uptake (WVU) was assayed using the following formula [64]:

WVU (%) = (Wt − Wi)/Wi × 100(2)

### 2.8. Gel Fraction

The in vitro evaluation of the gel fraction (GF) of the hydrogel membranes was conducted using the gravimetric method in PBS pH 7.4 at room temperature. Initially, the hydrogel membranes (1.5 cm diameter) were dried for 24–48 h at 55 °C until a constant weight was achieved (Wi). Next, the dried samples were immersed in excess PBS for a period of 24 h. After this immersion, the samples were removed from the liquid and dried again for 24–48 h at 55 °C to ensure complete evaporation of the solvent and weighted (Wf). To calculate the GF, the following formula was used [65]:

GF (%) = (Wf/Wi) × 100(3)

### 2.9. Degradation Study

The hydrogel membranes (1.5 cm in diameter) were dried for 24–48 h at 55 °C (Wi). Subsequently, the samples were immersed in PBS pH 7.4 at 37 °C for a period of 4 h. After this immersion, the samples were taken out of the liquid and dried for an additional 24–48 h at 55 °C (Wf). To assess the degradation rate (DR), the same samples underwent a second immersion in distilled water, this time for 20 h. The samples were finally dried for 24–48 h at 55 °C. The degradation rate was calculated using the provided formula [66]:

DR (%) = (Wi − Wf)/Wi × 100(4)

### 2.10. Dynamic Rheology Measurements

Oscillatory rheology investigation (frequency sweep tests) was performed on disk-shaped samples by employing a rheometer (GBC, VIC, Australia) equipped with a homemade jacket connected to a circulating water bath (Lauda Brinkman Ecoline E100, Suite C Marlton, NJ08053, USA) to ensure a constant temperature of 37.0 ± 0.1 °C during the measurements. A disk-shaped specimen was carefully placed between the two circular plates of the instrument oriented parallel to each other with a working gap between them corresponding to a relative compression of the sample of 10%. After a time period of 15 min of keeping the system at 37.0 ± 0.1 °C, the dynamic rheology measurements were carried out following a squeezing deformation induced by a motion of pseudorandom noise shape exerted on the sandwiched sample. Actually, this oscillatory displacement took place vertically by the upper plate with a very small amplitude compared to the value of the gap (thickness of the sandwiched specimen), giving rise to a corresponding force transmitted through the sample to the bottom plate below, where a very sensitive sensor recorded it continuously. The displacement and force data acquired during the measurements were processed by using a Fourier transform algorithm to finally give the values of the storage (G′) and loss (G″) moduli at 400 discrete frequencies concomitantly in the range of 0.25–100.00 Hz, with a step of 0.25 Hz. The dynamic rheological measurements were performed in triplicate (with a relative standard deviation of max. 15%), and every single rheogram was an averaged result of 30 consecutive scans acquired. The other operational parameters regarding the samples investigated and rheological measurements are listed in Table 2.

### 2.11. Antioxidant Activity

The constant-weight hydrogel samples (0.5 cm × 1 mm disks, approximatively 0.1 mg) were agitated in an orbital shaker at 200 rpm, 37 °C, in a solution of 1 mL 10^−6^ M DPPH (1 M in MeOH) diluted in ethanol/sample. The samples were incubated in the dark for 45 min. The absorbance of the samples was read at 517 nm against the control solution (the hydrogel sample free of biologically active compounds) [67]. The percentage of radicals scavenged can be determined using the following equation:

Radical scavenging activity (%) = [(Acontrol − Asample)/Acontrol] × 100(5)

### 2.12. Inhibition of Protein Denaturation

To investigate the anti-inflammatory properties of the hydrogels and their capacity to inhibit protein denaturation under pro-inflammatory conditions, we adopted a methodology based on Nowak’s work [68]. Circular hydrogel samples (1 × 1 × 0.1 cm) were used for the experiments, and the following formula was used:

Inhibition of denaturation (%) = (1 − At)/Ac,(6)

where At is the absorbance of the tested samples, and Ac represents the absorbance of the control samples at 280 nm.

The samples were immersed in a 5 mL solution containing 5% BSA (Sigma-Aldrich, USA) in PBS (pH = 7.4) and incubated at 200 rpm and 37 °C for 15 min. After this initial incubation, the samples were heated at 70 °C for 5 min. Subsequently, the reaction solutions were cooled using an ice bath to reach a temperature of 25 °C. For the control experiments, we used aspirin at a concentration of 0.5 mg/mL in PBS as the positive control and water as the negative control.

### 2.13. Doxycycline Release Study

#### 2.13.1. Cumulative Doxycycline Release

The in vitro release of doxycycline from the hydrogels was tested by adding a precise weight of the hydrogel samples (circular films with a diameter of 1 cm and thickness of 1.0 mm) to a suitable amount of synthetic wound fluid media with pH values of 7.4 and 8.3, respectively, to ensure the sink conditions. The experiments were conducted in an orbital shaker at 37 °C and 120 rpm. At 60-min intervals, 1.0 mL of the releasing media was collected, and the concentration of doxycycline in the collected samples was determined using a suitable calibration curve. The collected volume was replaced with an equal volume of fluid media at every sampling. This process monitored doxycycline release from hydrogel samples over time. The result is expressed as the percentage of cumulative drug released relative to the initial doxycycline concentration over time.

#### 2.13.2. Release Kinetic Study

The kinetic models applied are explained below:

Zero-order model
  Ct = C0 + K_0_t, (7)
  where Ct represents the drug amount dissolved at time t; C0 is the initial drug amount, and K_0_ is the zero-order release constant in concentration/time units. To analyze release kinetics, data from in vitro drug release studies were graphed as cumulative released drug amount against time [69].

First order model
  lnCt = lnC0 − K_1_t, (8)

This model describes drug absorption or elimination for certain drugs. The release of a drug with first-order kinetics is represented by Equation (8), where C0 is the initial drug concentration; C is the concentration at time t; K is the first-order rate constant, and t is time.

Higuchi model
  *Q* = *Kt*^0.5^ (9)

This correlation explains drug dissolution in diverse modified-release dosage forms, like specific transdermal systems and matrix tablets containing water-soluble drugs. The collected data were graphed as cumulative drug release percentage against the square root of time [69].

Korsmeyer–Peppas model
  Ct/C∞ = K × t^n^ (10)

To find out the mechanism of drug release, the first 60% of drug release data were fitted in the Korsmeyer–Peppas model, where Ct is the drug concentration in the release solution at time t; C∞ is the equilibrium concentration of the drug in the release solution; Ct/C∞ is a fraction of the drug released at time t, and K is the release rate constant, and n is the release exponent.

### 2.14. Antibacterial Activity

The antibacterial activity of hydrogel membranes containing npAg or DOXY was evaluated by an adapted protocol [70] against four Gram-positive bacteria (*S. epidermidis, S. aureus*, *E. faecalis*, *S. pyogenes*) and three Gram-negative bacteria (*P. aeruginosa*, *A. baumanii*, *K. pneumoniae*) employing disk diffusion test. This qualitative method is used to evaluate the antibacterial activity of samples employing zones of inhibition. First, bacterial cultures of the aforementioned strains were obtained and confirmed. Then, bacterial suspensions with 0.5 McFarland turbidity were prepared (1.5 × 10^8^ cfu/mL). The suspensions were then inoculated on suitable sterile agar media using the spread plate method to ensure even distribution. Next, pre-cut hydrogel membrane samples of 0.5 cm diameter were placed onto the inoculated agar surface. For this assay, pristine hydrogel membranes without any bioactive compounds were prepared to determine if the addition of collagen and hyaluronic acid had any impact on the membranes’ behavior (sample HM-Cp). Also, for the positive control, standardized doxycycline discs were used (30 μg). The plates were incubated at 37 °C for 24 h to allow for bacterial growth and potential interaction with the hydrogel membranes. After the incubation period, the zone of inhibition surrounding each sample was carefully evaluated and measured. The presence and size of the inhibition zone indicated the extent of antibacterial activity against tested strains, as described by CLSI guidelines (2016). The assay was conducted in duplicate.

### 2.15. Biocompatibility Study

#### 2.15.1. Protein Adsorption

The adsorption efficiency of the hydrogel membranes was assessed using BSA (5% *w*/*v* in PBS, pH 7) as the test protein, employing a method adapted from Gago et al. [71]. For the batch experiments, 5 mL of the BSA protein solution was added to circular hydrogel samples (1 × 1 × 0.1 cm). These samples were then placed in an orbital shaker operating at 200 rpm at 37 °C for 24 h. Using a calibration curve, both the initial and equilibrium protein concentrations were determined. Protein adsorption was assessed with a spectrophotometer (Tokyo, Japan) using the Bradford reagent and calculated using the provided equation:

q = (C_0_ − C_e_) × V/m, (11)

where q (mg g^−1^) is adsorption capacity; C_0_ and C_e_ (mg L^−1^) are the initial and equilibrium concentrations of protein in the solution, respectively; V (L) is the solution volume, and m (g) is the adsorbent mass.

#### 2.15.2. Cell Culture and Scratch-Wound Assay

Hs27 human fibroblasts (ATCC Crl1634) were routinely maintained in a cell culture incubator (PHC Europe, B.V., Nijverheidsweg, The Netherlands) at 37 °C and 5% CO_2_ atmosphere in DMEM supplemented with 10% fetal bovine serum. A total of 40,000 cells were incubated overnight in 96 well plates for full-surface coverage and scratched the next day with a 200 µL sterile tip. The scratch was documented by a full scan of each well using the EvosFL software v1.7. Each hydrogel sample was incubated in triplicates for 24 h, and the scan was repeated. The area measurements were made using the ImageJ plugin “Wound healing size tool” (available at https://github.com/AlejandraArnedo/Wound-healing-size-tool/wiki (accessed on 10 June 2023)). The nude area coverage was calculated as a percentage a from T0, with ImageJ software (version 1.8.0), using the following formula:

Coverage (%) = 100 × (nude area T0 − nude area T24)/nude area T0(12)

#### 2.15.3. Cell Proliferation Assay

The MTS assay was performed in the same wells tested for scratch-wound assay. After the 24 h image acquisition, the samples were removed, and the cell medium was replaced with 120 µL of phenol-free DMEM, supplemented with 10% fetal bovine serum and 6× MTS reagent. Cells were incubated (GC Life Science, Jaipur, India) for 3 h, and the color intensity was measured at 490 nm using a spectrophotometer (Anthos, GMBH, Austria). The results were calculated according to the following formula:

Viability (%) = 100 × [Experimental value (OD490) − background average (OD490)]/Mean value of the untreated cells (OD490)(13)

### 2.16. Surface Morphology

The morphological features of the biohybrid hydrogel membranes with and without antimicrobial agents were acquired using a Scanning Electron Microscope (FEI Company, Hillsboro, OR, USA) with an accelerating voltage of 5 and 10 kV. For the SEM images, all samples were coat-sputtered with Au to ensure the conductivity of the sample (60 s).

### 2.17. Statistical Analysis

Statistical analysis was performed using SPSS 26 (SPSS Inc., USA). More specifically, the experimental data for water vapor uptake, gel fraction, antioxidant activity, and anti-inflammatory activity were analyzed using ANOVA. Degradation rate data were analyzed using two-factor ANOVA. Data are presented as mean ± standard deviation (S.D.).

For the scratch-wound assay, statistical analysis was performed using GraphPad v7, One-Way ANOVA, and Dunnett multiple comparisons, where data were compared to the control.

## 3. Results and Discussion

### 3.1. Production of Biohybrid Hydrogel Membranes

The combination of natural and synthetic polymers in the production of wound dressings leads to a synergistic effect that enhances the material’s properties [72]. As shown in our previous work, polymer type, concentration, and the addition of bioactive compounds have the potential to significantly impact the properties of hydrogel membranes [60]. In this study, a combination of alginate and polyvinyl alcohol (PVA) was employed, alongside the inclusion of bioactive compounds like collagen and hyaluronic acid. Wound dressings can incorporate a wide range of antibiotics, such as vancomycin [73], tetracycline [74], erythromycin [75], ciprofloxacin [76,77], and gentamicin [78]. Our drug model of choice was doxycycline [7]. DOXY has been incorporated in nanofibrous membranes [79], hydrogel membranes and films [80,81], in situ gelling powder [82], microspheres [83], and sponges [84]. Metal nanoparticles have proven to be effective in enhancing the antimicrobial properties of wound dressings. They have been successfully incorporated into various scaffold materials like hydrogels [85] and nanofibers [86]. A crucial aspect when incorporating npAg into a wound dressing is to achieve a material that maintains strong antimicrobial activity while also exhibiting good biocompatibility and minimal cytotoxicity. In our methodology, we loaded the hydrogel membrane with the antimicrobial agents by directly mixing them into the formulation before the crosslinking process. Hydrogel materials are very hydrophilic, with a three-dimensional network structure that contains hydrophilic groups such as hydroxyl (−OH) or amine (−NH_2_) groups that may establish hydrogen bonds with water molecules and other hydrophilic molecules such as doxycycline. As a result, the molecular structure of hydrogel membranes that aid in doxycycline retention is frequently based on the network of polymeric chains of the hydrogel. In addition, the polymeric hydrogen chains form a network that can physically entrap doxycycline molecules and prevent them from diffusing out of the hydrogel when it is not hydrated. Furthermore, electrostatic interactions between the charged functional groups of the polymer and the doxycycline molecules can aid retention. To avoid potential undesired byproducts that might arise from the reaction and better control of the final concentration of the npAg, we choose to use ex situ-synthesized nanoparticles [87]. The npAg can also interact with hydroxyl and amine functional groups available in the matrix, establish electrostatic interactions, and sit into the pores of the foamed network [88,89].

### 3.2. Macroscopic Evaluation and pH

**Figure 2:**
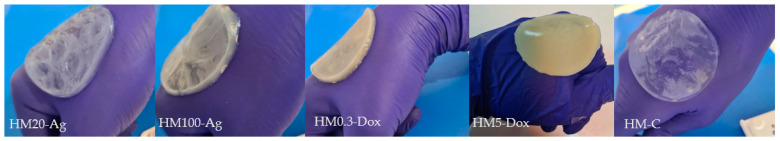
Visual representation of samples.

Following the completion of the crosslinking protocol, all samples were formed into the hydrogel membranes, and a macroscopic analysis was conducted. All samples exhibited a uniform and homogenous appearance. However, the surface of the hydrogel membranes displayed some irregularities and had impressions of crystals, likely attributed to the freeze–thaw cycles utilized during the production process. The color of the hydrogel membranes varied depending on the integrated compound, as visually depicted in Figure 2. Except for sample HM5-Dox, which contained a high concentration of DOXY and appeared dark yellow and opaque, the rest of the samples were translucent, while the control sample was transparent. The samples demonstrated favorable physical characteristics and the ability to adapt to the body curves. Each sample had a specific odor, mostly due to the addition of alginate. Moreover, all samples exhibited some degree of skin adhesion and provided a pleasant cooling sensation upon contact with intact skin. Table 3 shows information about weight and thickness variation, as well as pH. The pH values of the samples ranged from 4.62 ± 0.03–7.34 ± 0.05. Notably, samples containing DOXY exhibited lower pH values, with sample HM5-Dox being the most acidic (pH 4.62 ± 0.03). This result can be attributed to the acidic nature of the DOXY solution that was added at a higher concentration. For reference, a 2% stock solution of DOXY in distilled water showed a pH value of 2.26, while the blank hydrogel membrane (sample HM-C) had a pH value of 7.34 ± 0.05. The literature also reported similar findings, demonstrating that the incorporation of DOXY resulted in a reduction in the pH values in the samples [80]. Numerous research papers have investigated the pH of the wound environment and sought to identify the most effective pH for wound dressings, considering the unique characteristics of each wound. Depending on the type of wound, both neutral and acidic pH wound dressings are considered appropriate [90]. Sample HM5-Dox, with its high DOXY concentration, would be suitable for treating chronic and complicated wounds that often exhibit high pH values, making its acidic pH beneficial in this context.

### 3.3. Swelling Index

**Figure 3:**
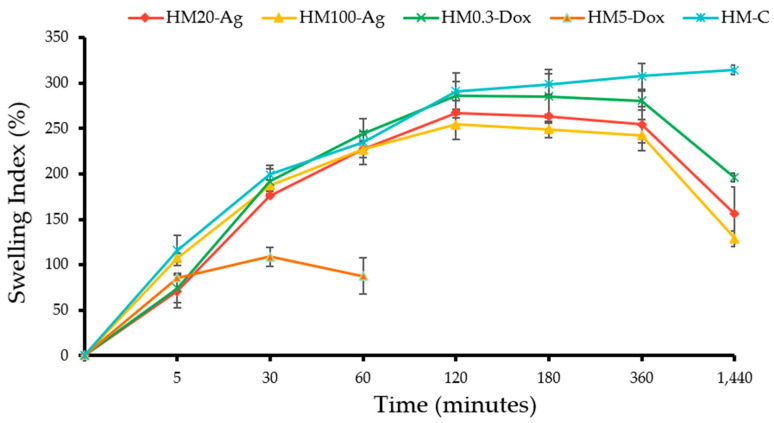
Swelling index of the hydrogel membranes. Samples were analyzed in triplicate, and the results are presented as mean ± S.D.

The addition of both npAg and DOXY influenced the swelling behavior of the hydrogel membranes compared to the control (Figure 3). For samples HM20-Ag and HM100-Ag, the maximum swelling index was at 2 h, reaching 267.23 ± 1.96 and 254.29 ± 7.31%, respectively. Between 2 and 6 h, these samples showed an equilibrium state. However, after 6 h, the matrix began to solubilize, which led to the disruption of its water retention capacity. The presence of both 20 nm and 100 nm npAg decreased the swelling ability of the hydrogel membranes, with no significant difference observed based on nanoparticle size. These results align with findings from the existing literature [87,91,92,93] as well as with the other results obtained within this study, where the addition of npAg has been shown to diminish the crosslinking density of polymer chains and concurrently decrease the hydrophilicity of the hydrogel due to the hydrophobic nature of nanoparticles. It is worth mentioning that some studies have reported contrasting outcomes, highlighting an increase in the swelling ability upon the introduction of npAg. These results can be attributed to pore enlargement that led to superior water-holding capacity [94,95] or increased electrostatic repulsion between ionic charges in the polymer network [96]. The outcome often depends on the properties and concentrations of the nanoparticles, which may or may not impact the swelling behavior of the hydrogel [85].

The addition of DOXY influenced the swelling behavior in a concentration-dependent manner (Figure 3). Sample HM0.3-Dox showed maximum swelling at 2 h, reaching a value of 285.68 ± 6.99%. However, beyond the 6 h timeframe, the hydrogel matrix started to solubilize, leading to a gradual decline in its water-holding ability. When compared to all other specimens, sample HM0.3-Dox exhibited the most remarkable swelling performance, similar to that of the control. Conversely, in the case of sample HM5-Dox, the introduction of a high concentration of DOXY yielded an adverse effect on the swelling ability. At 30 min, the swelling index was 108.77 ± 8.61%, followed by a rapid destructuring of the hydrogel matrix at 1 h. This instability culminated in compromised structural integrity of the sample after only 2 h, making adherence to the test protocol impossible. Hedayatyanfard et al. reported that the addition of genipin and doxycycline reduced the water uptake ability of semi-IPN films based on chitosan/PVA [81]. Patlolla et al. found that the addition of doxycycline to buccal films based on chitosan, gelatin, and Eudragit^®^ RS led to slower water uptake [97]. We presume that the addition of DOXY can influence the hydrogel’s properties and swelling behavior due to the high number of functional groups, which could potentially disrupt the crosslinking of polymer chains within the hydrogel matrix. Also, several other factors, such as molecular size, ionic interactions, and hydrophobic–hydrophilic interactions, could potentially influence the water uptake ability.

### 3.4. Water Vapor Uptake

**Figure 4:**
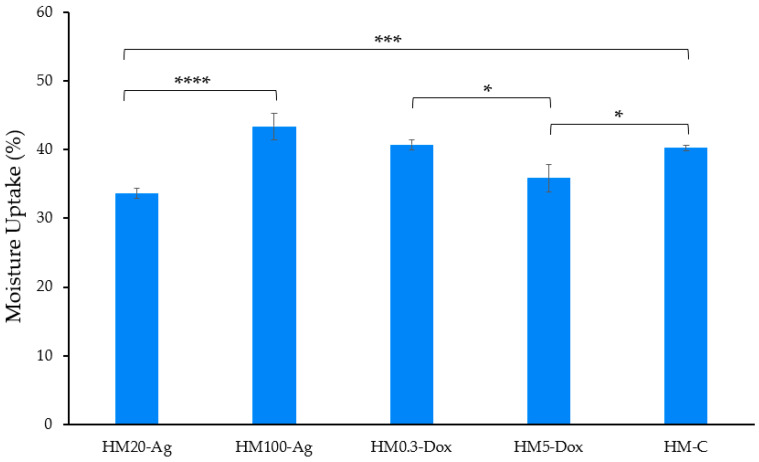
Moisture uptake of hydrogel membranes. Samples were analyzed in triplicate, and the results are presented as mean ± S.D, where **** *p* < 0.0001; *** *p* < 0.001; and * *p* < 0.05.

The moisture uptake values were found to be significantly different between the sample groups (*p* < 0.0001). Sample HM20-Ag exhibited the lowest moisture uptake capacity at 33.36 ± 0.73%, while sample HM100-Ag had the highest moisture capacity of 43.39 ± 1.91% (Figure 4) among all samples. The incorporation of smaller-sized npAg (20 nm) resulted in a reduction in the moisture uptake capacity when compared to the inclusion of 100 nm npAg and the blank hydrogel membrane. Similarly, the introduction of a higher dose of DOXY led to a decrease in moisture uptake ability when compared to the low-dose DOXY and the blank hydrogel membrane. These findings suggest that small-sized nanoparticles and a high dose of DOXY have a negative impact on the moisture uptake capacity of the hydrogel membranes. The addition of nanoparticles can influence the hydrogel’s properties, and this is largely due to the unique characteristics of nanoparticles [67,98], such as their high surface area and their ability to interact with pores [99,100]. Also, the presence of DOXY with its many functional groups [101] can influence the properties of the hydrogels by affecting the formation of bonds between the polymer chains and their ability to attract and retain water.

### 3.5. Gel Fraction

**Figure 5:**
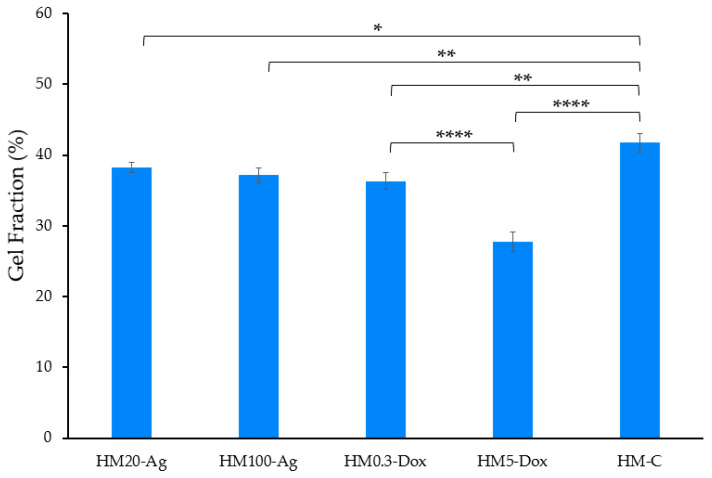
Gel fraction of the hydrogel membranes. Samples were analyzed in triplicate, and the results are presented as mean ± S.D. where **** *p* < 0.0001, ** *p* < 0.01, and * *p* < 0.05.

The results revealed a significant difference in gel fraction values among the sample groups (*p* < 0.0001). Sample HM-C had the highest gel fraction value of 41.73 ± 1.32%, while sample HM5-Dox had the lowest value of only 27.71 ± 1.4% (Figure 5). The inclusion of 20 and 100 nm npAg, as well as a low dose of DOXY, resulted in a slight decrease in the gel fraction compared to the control. However, the addition of a higher dose of DOXY significantly reduced the gel fraction. While there was not a notable reduction in gel fraction among samples containing npAg, there was a significant difference in gel fraction between samples with low and high DOXY doses. Alcântara et al. showed that the addition of silver to hydrogels obtained by ionizing radiation had little impact on the gel fraction [102]. Boonkaew et al. found that incorporating silver nitrate led to comparable gelation of the hydrogels obtained using the UV radiation technique [92]. Popescu et al. reported that the gel fraction of a hydrogel obtained by the freeze–thaw method is influenced by the concentration of chitosan-capped npAg, where higher npAg concentrations resulted in lower gel fractions [103]. We can presume that the incorporation of silver intro hydrogels may influence the formation of bonds between polymer chains, and this effect could vary based on the properties and concentration of npAg as well as the crosslinking method employed [104,105]. Due to the presence of an increased number of DOXY molecules, which possess many functional groups, the formation of non-covalent interactions between the polymer chains was hindered, resulting in a reduced gel fraction value. Wassenaar et al. demonstrated that in a PVA/alginate hydrogel produced via the freeze–thaw method, variation in polymer ratio was not the sole influencing factor of the gel fraction; the introduction of sodium ampicillin also contributed to a reduction in this value [106].

### 3.6. Degradation Study

**Figure 6:**
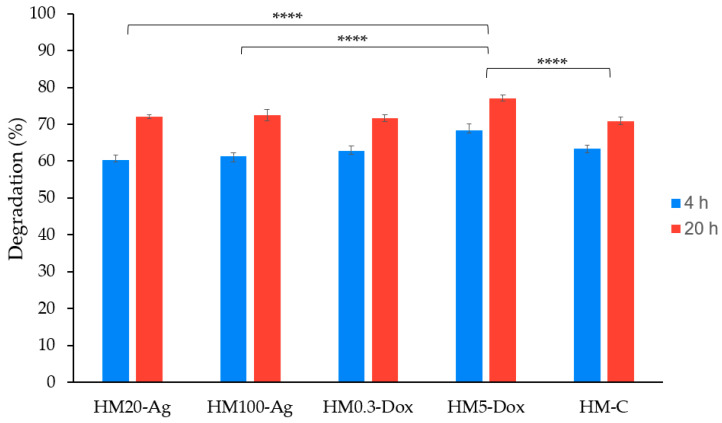
In vitro degradation study of hydrogel membranes at 4 and at 20 h. Samples were analyzed in triplicate, and the results are presented as mean ± S.D. where **** *p* < 0.0001.

The degradation rate of the samples ranged from 60.29 ± 1.38 to 68.46 ± 1.66% at 4 h and between 70.88 ± 1.07 and 77.10 ± 0.86% at 20 h (Figure 6). The degradation rates differ significantly between the hydrogel membranes (*p* < 0.0001). Degradation increased significantly from 4 h to 20 h across all samples (*p* < 0.0001). The change in degradation over time varies depending on the formulation (*p* < 0.05). The samples that contain npAg tend to have a slight reduction in the degradation rate compared to the control at 4 h but show a higher degradation rate compared to the control at 20 h, which is not statistically significant. Sample HM5-Dox consistently showed higher degradation compared to the other samples at both 4 h (68.46 ± 1.66%) and 20 h (77.10 ± 0.86%). All other comparisons between samples at 4 and 20 h are not statistically significant (*p* > 0.05). At both 4 h and 20 h, there was no significant difference in degradation between HM20-Ag and HM100-Ag, as well as between HM0.3-Dox and HM5-Dox (*p* > 0.05). These results are consistent with the gel fraction findings, suggesting a correlation between gel fraction and degradation rate, as expected. Samadi et al. showed that the addition of npAg to a glycerol/chitosan/polyvinyl alcohol-based hydrogel significantly decreased the degradation rate [91], while Qui et al. showed that an increase in npAg concentration in a hydroxypropyl methylcellulose-hydroxyapatite scaffold hydrogel leads to an increased degradation rate [105]. Wassenaar et al. investigated how DOXY incorporation affects the degradation rate of an injectable hydrogel derived from decellularized porcine ventricular myocardium. The modulation of degradation rate was evident in reduced collagenase degradation in vitro and diminished matrix degradation in vivo through MMP inhibition, while the hydrogel maintained biocompatibility and mechanical properties [107].

### 3.7. Oscillatory Rheology—Frequency Sweep Tests

The investigated hydrogel membranes undergo small squeezing deformations (around 0.005%), leading to corresponding low-amplitude oscillatory shear strain (SAOS). Dynamic rheology data reveal two distinct viscoelastic states, a gel-like state (G′ > G″, frequency-independent) and a liquid-like state (G′ < G″, frequency-dependent). The crossover point, where G′ equals G″, defines a gel point. G′ represents stiffness and energy storage, while G″ indicates viscous behavior and energy dissipation as heat. This distinction is characterized by the loss tangent (tgδ) with values <1 for gel-like behavior, >1 for liquid-like behavior, and =1 at the crossover point [108,109,110,111].

**Figure 7:**
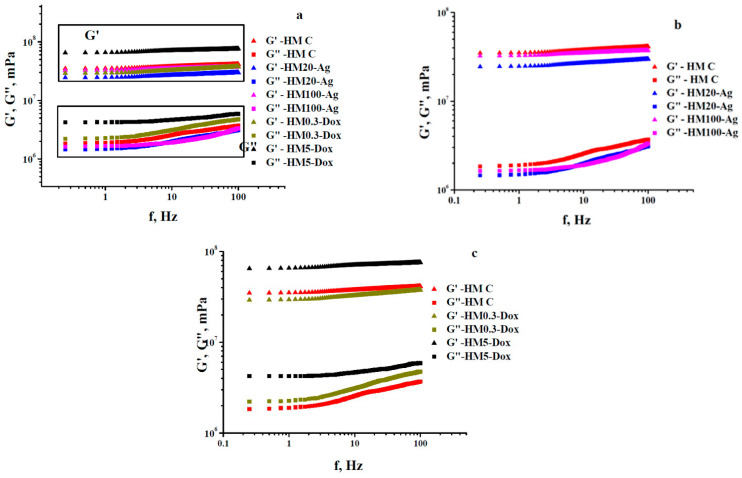
Frequency evolution of viscoelastic moduli acquired at 37 °C: (**a**) general picture outlined for all five systems; (**b**) comparative plots for hydrogels HM20-Ag and HM100-Ag taken in relation to those for control hydrogel; (**c**) comparative rheograms for hydrogels HM0.3-Dox and HM5-Dox compared to the control sample.

**Figure 8:**
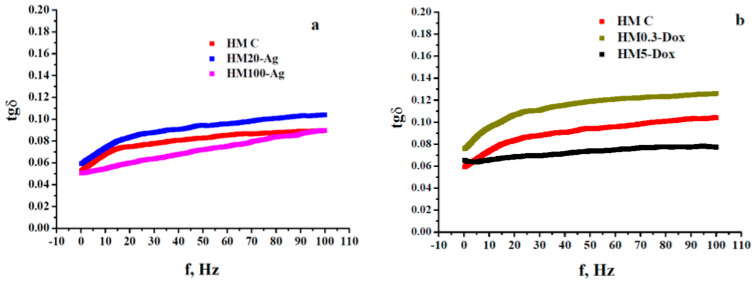
Comparative variation in loss tangent as a function of frequency at 37 °C for the specified hydrogels: (**a**) comparation between samples HM-C, HM20-Ag and HM100-Ag; (**b**) comparation between samples HM-C, HM0.3-Dox and HM5-Dox.

The frequency-seep tests carried out on the investigated systems revealed their strong gel-like properties, with G′ values larger than those of G″ by at least one order of magnitude over the operational frequency range of 0.25–100 Hz. This well-defined behavior can be noticed in Figure 7. First of all, a comparative analysis of the rheological findings for the samples containing npAg displays quite a different behavior if considered G′-f rheograms and very similar properties when accounted for G″-f dependences (Figure 7a). Thus, taking them together, the presence of 100 nm npAg seems to strengthen the gel-like character (sample HM100-Ag) by comparison with the hydrogel containing 20 nm npAg (sample HM20-Ag). In practical terms, sample HM100-Ag (having the same rigidity as the control one) is stiffer than sample HM20-Ag, but both show a gel-like behavior following the order relationship between G′ values (Figure 7b), as mirrored by the values of loss tangent graphically plotted in Figure 8a: sample HM100-Ag is stronger (and stiffer) than sample HM20-Ag (more flexible), which is equivalent to say that the values of tgδ for HM100-Ag are generally smaller than those calculated for HM20-Ag. In this case, to partially conclude, the smaller npAg size increased the hydrogel flexibility compared to that of the control hydrogel, and adding the larger npAg maintained almost the same stiffness of the final hydrogels as for that of the control membranes, even though the gel-like character (the elastic component is by far prevalent concerning the dissipative energy/loss component) was strong for all three gels (tgδ < 0.1 for f ∈ [0.25, 100 Hz], Figure 8a).

As far as the hydrogels with DOXY are concerned, the data of dynamic rheology obtained at the same temperature of 37 °C showed a viscoelastic behavior different from that exhibited by the membranes with npAg by considering the same kind of comparative analysis made toward the viscoelasticity of the control membranes. As presented in Figure 7c, a low-dose DOXY (0.3 mg/mL) resulted in the hydrogel (HM0.3-Dox) exhibiting almost the same rigidity (as indicated by the G′ values) as that of the control hydrogel (sample HM-C). Conversely, a significantly higher doxycycline content (5 mg/mL) led to increased stiffness in the samples (HM5-Dox), surpassing both HM0.3-Dox and control membranes. At the same time, a similar evolution was observed for the viscous/dissipative energy character (accounting for G″ values): the larger the content of DOXY, the higher the loss modulus values. Moreover, the two G″-f rheograms of the two doxycycline-containing hydrogels are located above that of the control hydrogels, as shown in graphs in Figure 7c. However, all these three systems displayed a pronounced gel-like character if taking into account the much smaller than unity values of loss tangent (tgδ < 0.13, Figure 8b). The enhancement of gel-like character (and also hydrogel rigidity) at higher DOXY concentrations could be related to a possible crosslinking ability of such a molecular structure via attractive interactions with some components of the hydrogel network.

**Figure 9:**
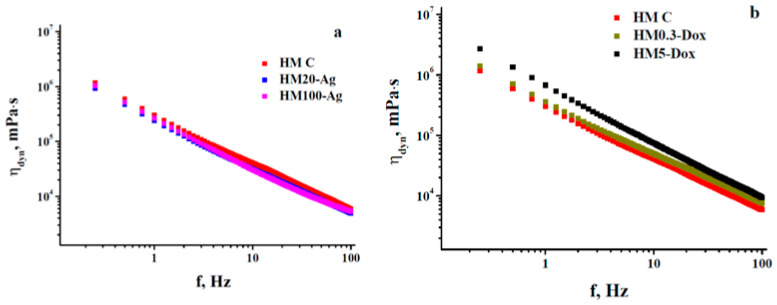
Frequency-dependences of dynamic viscosity measured at 37 °C for hydrogels (**a**) HM20-Ag—HM100-Ag and (**b**) HM0.3-Dox—HM5-Dox considered in comparison with that of the control sample.

The dissipative energy properties as a part of the viscoelastic behavior of the investigated hydrogels may be properly assessed by obtaining the values of dynamic viscosity (η*_dyn_* = *G″*/*ω*, where *ω* is the angular frequency (in rad/s) defined as *2∙π∙f* [110,112]) as a function of the frequency used during the frequency-sweep trials. The plots inserted into Figure 9a,b illustrate the same order relationships established between the rheograms G″-f shown in Figure 7, but with a substantial decrease in vertical shifting between them. Nevertheless, for all these strong enough hydrogels (G′ >> G″) for which the flow process is actually frozen during deformation, it is not advisable/practical to make an analysis based on viscosity dependences (mandatorily involving a flow phenomenon) because such an approach has a high degree of redundancy [110].

**Figure 10:**
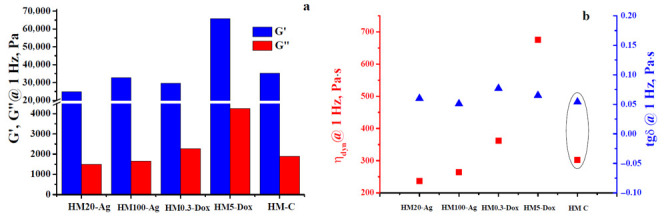
Some rheological parameters taken at 1 Hz and 37 °C comparatively displayed for the investigated hydrogels.

The usefulness of the rheological data acquired during frequency-sweep trials at a certain temperature resides in describing the viscoelastic behavior of the hydrogels employed to mimic conditions at rest (low frequencies, Figure 10) or those under stress (different higher frequencies, Figure 7, Figure 8 and Figure 9).

### 3.8. Antioxidant Activity

**Figure 11:**
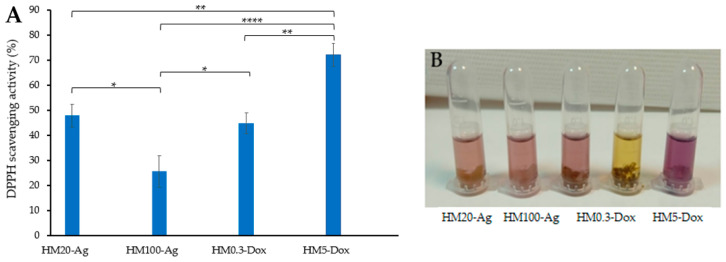
(**A**) DPPH scavenging activity of hydrogels after incubating in the dark for 45 min. (**B**) Visual representation of color changes after incubating the samples in the dark for 45 min. The samples were analyzed in triplicate, and the results in (**A**) are presented as mean ± S.D, where **** *p* < 0.0001; ** *p* < 0.01, and * *p* < 0.05.

The antioxidant activity of hydrogels was monitored using a 2,2-diphenyl-1-picrylhydrazyl (DPPH) assay [113]. The results indicate that there is a significant difference in antioxidant activity levels among the samples (*p* < 0.0001). The inclusion of 20 nm npAg positively influenced the antioxidant activity of the hydrogel membranes compared to the addition of 100 nm npAg. Similarly, hydrogel membranes containing a higher dose of DOXY exhibited greater antioxidant activity compared to those with a low dose of DOXY. Interestingly, sample HM5-Dox displayed significantly higher antioxidant activity compared to all other samples. This indicates that HM5-Dox may have potential benefits in terms of antioxidant properties. The experimental results of the tested hydrogels align well with the findings in the literature [114], particularly highlighting the notable scavenger activity exhibited by DOXY (attributed to the presence of hydroxyl groups), even when it is integrated within a matrix (Figure 11). Kharat and Mendhulkar assessed synthesized nanoparticles’ antioxidant activity using the DPPH assay, noting their potential as radical scavengers [115]. Priya et al. studied the antioxidant activity of nanoparticles from *P. pinnata* extract, demonstrating their scavenging potential [116]. Patra and Baek demonstrated robust antioxidant activity in terms of DPPH radical scavenging of npAg obtained from aqueous plant extracts [117]. These findings strongly suggest that npAg could serve as natural antioxidants for safeguarding health against oxidative stress [118]. In the same line of observations, the hydrogel formulation enriched with silver nanoparticles presents anti-scavenging activity, dependent on the size of the npAg.

### 3.9. Anti-Inflammatory Activity

**Figure 12:**
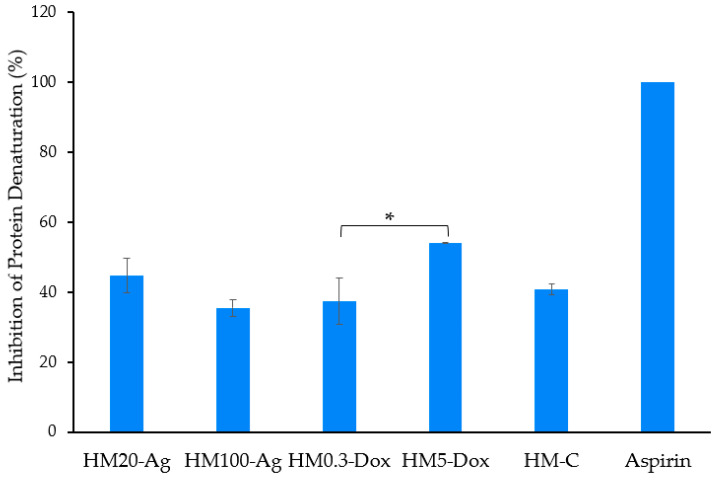
Inhibition of protein denaturation. The hydrogel samples were immersed in a 5% BSA solution at 37 °C for 15 min, then at 70 °C for 70 min, followed by cooling to 25 °C. The remaining BSA solution’s absorbance was measured at 280 nm, using aspirin solution (0.5 mg/mL) as control. The results are presented as mean ± S.D.; n = 3, where * *p* < 0.05.

It has been shown that preventing protein denaturation by medicinal agents in vitro indicates an anti-inflammatory effect. The assays used BSA as a model protein and aspirin as a positive control, and determinations were performed at 280 nm [60]. It was observed that all hydrogel formulations present an anti-inflammatory activity, protecting BSA from denaturation (Figure 12). The results indicate that there was a significant difference in the inhibition of protein denaturation among samples (*p* < 0.0001). The sample HM0.3-Dox (37.38 ± 8.1%) showed lower inhibition of protein denaturation compared to HM5-Dox (*p* < 0.05). Based on these results, we can conclude that the addition of DOXY in a higher concentration had a positive effect on the antioxidant effect. However, the results show that the antioxidant effect is minimally influenced by the addition of npAg or DOXY in low doses, and that is most likely attributed to the hydrogel matrix formulation, as there is no statistically significant difference between the sample HM-C and the rest of the samples (*p* > 0.05).

### 3.10. Doxycycline Release Study

#### 3.10.1. Cumulative Doxycycline Release

Hydrogels are classified based on their drug release mechanism into diffusion-controlled, swelling-controlled, chemically-controlled, and environmentally responsive systems [119]. The drug’s release profile and kinetics are crucial as they link in vitro and in vivo drug responses. UV-Vis spectrophotometry at λmax = 346 nm was used to assess the cumulative release of DOXY from the alginate/PVA/COL/HA hydrogel membranes during dissolution. In the domain of hydrogel membranes and drug delivery systems, upholding sink conditions is paramount during in vitro dissolution testing. Sink conditions help ensure that the release of the drug from the hydrogel membrane is not limited by the drug’s solubility in the surrounding medium. We checked some considerations for maintaining sink conditions in the context of hydrogel membranes: (1) High dissolution medium volume relative to the amount of drug released was 50:1 volume of dissolution medium to the amount of drug; (2) Frequent sampling of the dissolution medium that allows for monitoring of the drug concentration over time. An amount of 1 mL of the release medium was sampled at specific time points, and an equal volume of fresh simulated wound fluid was replaced to maintain a sink condition; (3) Low drug dose in the hydrogel membrane relative to the volume of the dissolution medium to avoid saturation of the medium. Our results are presented as cumulative drug release percentage from the initial concentration in hydrogel versus time.

**Figure 13:**
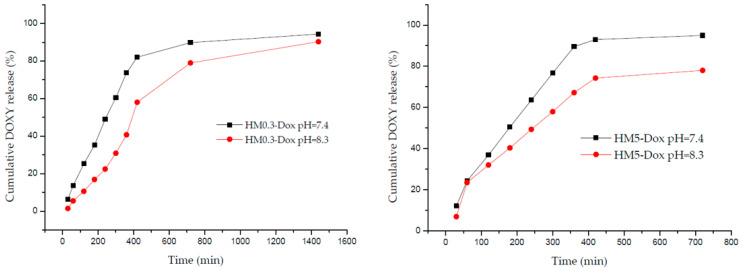
Cumulative doxycycline release vs. time. The results are presented as mean; n = 3.

The data from Figure 13 highlight notable trends in DOXY release from the hydrogels. For hydrogels loaded with DOXY in low dose (sample HM0.3-Dox), there was a substantial 90% release within the initial 12 h at pH 7.4 and a slightly reduced but still significant 80% release after the same duration at pH 8.3. In the case of hydrogels with a higher DOXY concentration (sample HM5-Dox), we observed a rapid release, with 90% achieved within 6 h in synthetic wound fluid media at pH 7.4. Furthermore, sample HM5-Dox exhibited a maximum of 80% release at the 12-h mark, pH 8.3. Beyond this point, samples HM5-Dox disintegrate, preventing further release study. At pH 8.3, both the rate and total percentage of drug released are slightly lower, indicating a delayed and modulated release profile. These outcomes align with the observations from the degradation and swelling assays. Specifically, sample HM0.3-Dox displayed a swelling profile similar to that of sample HM-C and a degradation rate not significantly different from all other samples. Conversely, samples containing high-dose DOXY exhibited a rapid disintegration rate compared to all other samples, which was also evident in the swelling assay, where extensive manipulation led to sample destructuring after only 1 h. These results suggest that the release of DOXY from the hydrogel matrix is influenced by a combination of factors, including drug concentration, pH, and structural stability of the hydrogel membrane.

#### 3.10.2. Release Kinetic Study

**Figure A1:**
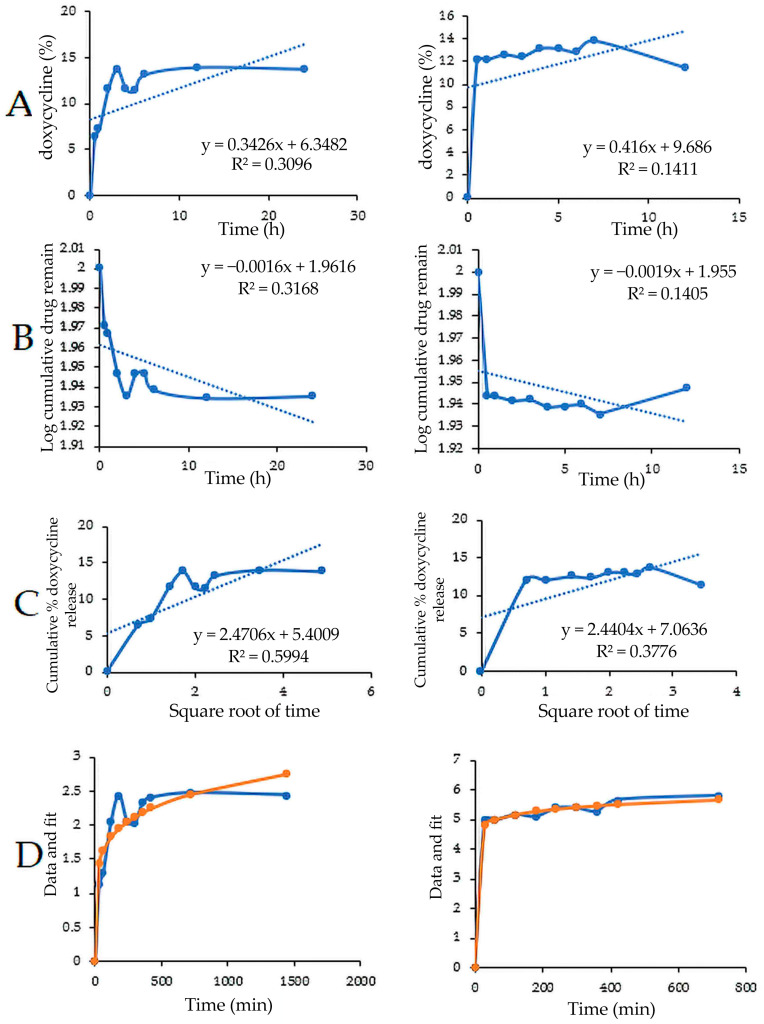
(**A**) Zero-order kinetics. Cumulative drug release vs. time. (**B**) First-order kinetics. Log cumulative drug remain vs. time. (**C**) Higuchi model. Cumulative % doxycycline release vs. square root of time. (**D**) Korsmeyer–Peppas model. Data and fit vs. time. For samples HM0.3-Dox (**left**) and HM5-Dox (**right**).

Kinetic models were applied to the data, and the goodness of fit was determined by comparing experimental and model data, indicated by the regression coefficient (R^2^). The most appropriate fit is suggested by R^2^ values closely aligned with the experimental data [120]. We utilized various kinetic models, including zero order, first order, Higuchi, and Korsmeyer–Peppas, in our approach (see Appendix A, Figure A1). The Korsmeyer–Peppas model, also known as the Power Law model, is useful for complex situations involving multiple kinetics, like drug release from hydrogels. To evaluate the mechanism of drug release from hydrogels, data for the first 60% of drug release were plotted in the Korsmeyer equation as log cumulative percentage of drug released vs. log time, and the exponent n was calculated through the slope of the straight line. The value of n characterizes the release mechanism of drugs, and this approach is particularly relevant when processes involve a combination of release mechanisms, such as Fickian diffusion (when the release exponent n is 0.5) and non-Fickian transport (when 0.5 < n < 1) [61,69,121].

The release mechanism of DOXY from the hydrogel membranes is attributed to the swelling properties of the hydrogels. As water molecules penetrate the polymer network, the drug undergoes diffusion and dissolution in the aqueous environment of the wound exudates. By observing the fractional release of DOXY at specific time intervals, we can assess the total amount of drug released from the hydrogel. To enable drug diffusion within the hydrogel, the polymer chains need to relax initially. The release of doxycycline from hydrogels is influenced by two parallel processes: water penetrating the hydrogel; and the drug diffusing through the expanding hydrogel [15]. According to Peppas, the release of drugs from controlled-release systems like PVA matrices exhibits exponential dependence. The drug release hinges on factors such as matrix swelling followed by drug diffusion, matrix breakdown followed by drug diffusion, and external triggers (like temperature or pH changes) that alter matrix properties and lead to subsequent drug release. The cumulative release of doxycycline hyclate from both samples (low and high concentration DOXY) at 37 °C, pH 7.4, is illustrated in Figure 13. We assessed the percentage of DOXY from the initial amount released at different times and compared the results using different kinetic models, which are listed in Table 4.

The release data analyzed based on Korsmeyer Peppas kinetics give the highest correlation coefficients, as can be seen in Table 4, indicating that the mixture of PVA and alginate polymers is suitable for formulating a hydrogel membrane that can be used as a drug delivery system. For both samples, the value of n indicates a non-Fickininan dissolution process governed by an anomalous diffusion mechanism or diffusion coupled with erosion.

### 3.11. Antimicrobial Analysis

**Figure 14:**
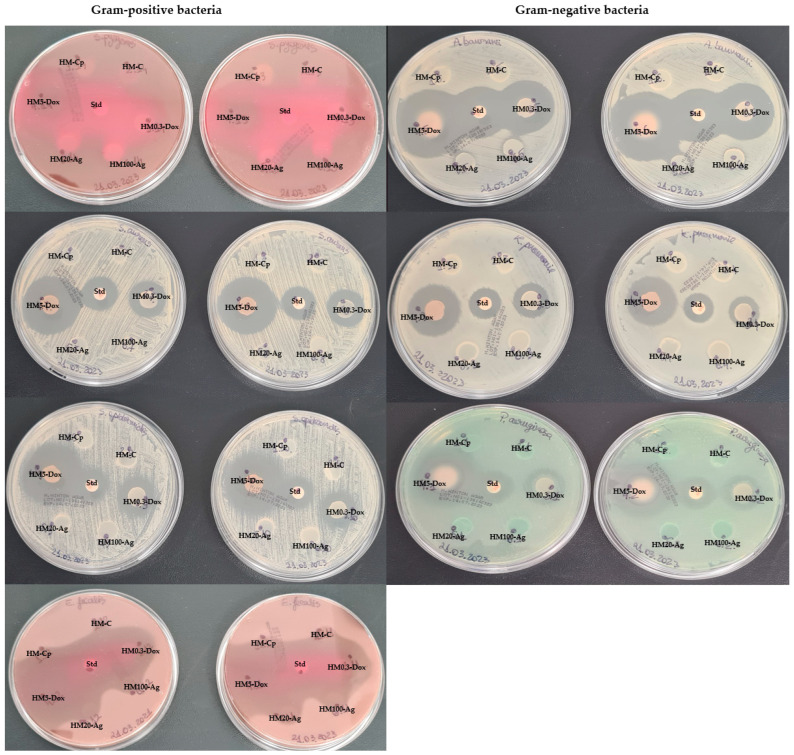
Inhibition zones for different microbial strains (duplicate).

In our work, we have selected seven microbial species, both Gram-positive and Gram-negative, to assess our hydrogel membrane’s antimicrobial activity and optimal drug concentrations, providing a comprehensive evaluation for potential applications (Figure 14). The samples HM-C and HM-Cp were considered negative controls due to their lack of intrinsic antibacterial activity. The samples HM0.3-Dox and HM5-Dox exhibited a clear zone of inhibition for both Gram-positive and Gram-negative bacteria. The dimension of the inhibition zone was found to be concentration-dependent, as expected (Figure 14) [7,81,82,122]. Notably, the sample HM0.3-Dox demonstrated an inhibition zone comparable to that of the standard DOXY disc for *S. epidermidis*, *E. faecalis*, *S. pyogenes*, *P. aeruginosa*, and *K. pneumoniae.* The sample HM5-Dox exhibited a significantly larger inhibition zone in comparison to sample HM0.3-Dox for all tested microorganisms, as expected. Our results show that the incorporation of DOXY at both low dose and high dose was successful and that DOXY maintained its clinical effect with successful release. In contrast, the samples HM20-Ag and HM-100-Ag did not display any inhibition zone for any of the tested bacterial strains (Figure 14). Recent studies reported similar results when using disk diffusion assay [123], but by using a different technique (broth dilution), the results showed that the hydrogels containing npAg had antimicrobial activity. However, the results were inconsistent for the different types of formulations [102]. The antimicrobial activity of npAg could be attributed to several factors, such as the concentration [93,124,125] and properties [94,125] of the npAg and release kinetics of the silver ions [93,124], as well as the number of microbial cells [126]. The lack of an inhibition zone might be attributed to the limited release of nanoparticles caused by the presence of polymers on their surface [127,128], which restricts their diffusion. Additionally, the stability of the silver ions (Ag^+^) could play a role in this phenomenon [102]. At lower concentrations, silver ions could potentially interact with the -COOH functional groups present in alginate, resulting in a reduction in the antibacterial effect [129]. Furthermore, the hydrogel discs exhibited some swelling during the 24 h assay. As a result, the absence of an inhibition zone could be attributed to the coverage caused by the swelling effect, as sometimes the inhibition zone is rather small, only a couple of mm [125,130]. The measured inhibition zones (in mm) for each sample are provided in Table 5. Upon comparing the measured inhibition zones to Clinical and Laboratory Standard Institute (CLSI) guidelines (2016), it was concluded that hydrogel membranes incorporating DOXY possessed significant antibacterial activity (see Appendix A, Table A1). These findings, in conjunction with the drug release assay, support the idea that the hydrogel membranes obtained in this study effectively and optimally encapsulate and release the antimicrobial agent. Our results are similar or even superior to those reported in the existing literature for other drug delivery scaffolds [122,131,132,133]. A recent study (2019) highlighted that the nanofiber scaffold had lost the antibacterial activity compared to the film scaffold for *P. aeruginosa*, emphasizing the importance of scaffold selection for DOXY topical delivery [81]. Wound contamination or infection presents a challenge that hinders the healing process and gives rise to further complications [134,135,136]. The ability of wound dressings to encapsulate and release antibacterial agents is a highly desirable attribute as it not only supports wound healing but also helps prevent and treat complications [15,137]. Despite the absence of an inhibition zone in samples HM20-Ag and HM100-Ag, we observed that the hydrogel membranes incorporating npAg exhibited a notable advantage: improved storage stability at room temperature. While pristine hydrogel membranes (sample HM-Cp) demonstrated satisfactory stability when stored at room temperature, the inclusion of COL and HA (sample HM-C) led to rapid contamination of the samples under the same conditions. In contrast, samples HM20-Ag and HM100-Ag, which contained both COL, HA, and npAg, exhibited prolonged storage stability at room temperature without contamination. This observation is consistent with previously reported findings, which indicate that hydrogels incorporating npAg possess an antiseptic activity [88,127,138]. The storage stability at room temperature for samples containing DOXY was found to be satisfactory, with no contamination.

### 3.12. Biocompatibility Study

#### 3.12.1. Protein Adsorption

**Figure 15:**
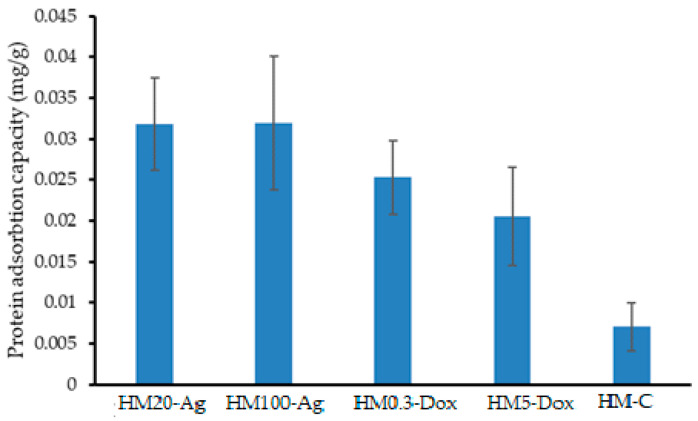
Protein adsorption capacity. The remaining BSA post-hydrogel removal was measured via the Bradford method. Protein uptake after 24 h was indirectly assessed, factoring in initial and equilibrium BSA concentrations, plus sample dimensions. Outcomes are shown as mean ± S.D.

Protein adsorption onto hydrogels is a significant phenomenon with implications in various fields, such as biomaterials, drug delivery, tissue engineering, and medical devices [139]. The adsorption mechanisms could be attributed to electrostatic interactions, hydrogen bonding, hydrophobic interactions, and specific binding sites on the hydrogel surface [140]. Protein adhesion plays a major role in determining the biocompatibility of materials and can lead to the formation of a protein layer on the hydrogel surface, often referred to as a “biofouling” layer. This adsorbed protein layer is often referred to as the “protein corona” [141]. This layer can impact the interactions between the hydrogel and the surrounding biological environment, affecting the hydrogel’s biocompatibility, stability, and functionality. A low protein absorption on hydrogels is beneficial for the systems used in contact with human fluids because it prevents the accumulation of proteins that can promote microbial growth, reducing the risk of secondary infection. Our findings show that all hydrogel samples present low-protein adsorption (Figure 15). However, the sample HM-C shows the least adsorption capacity compared to the rest of the samples. The different behavior is due to the presence of the polar ionized groups of DOXY from the hydrogel matrix that allows for higher adsorption of BSA on the surface of the hydrogels via electrostatic interactions. Also, we supposed that some silver nanoparticles are ionized in the hydrogel matrix and enhanced these interactions.

#### 3.12.2. Cell Proliferation Assay

**Figure 16:**
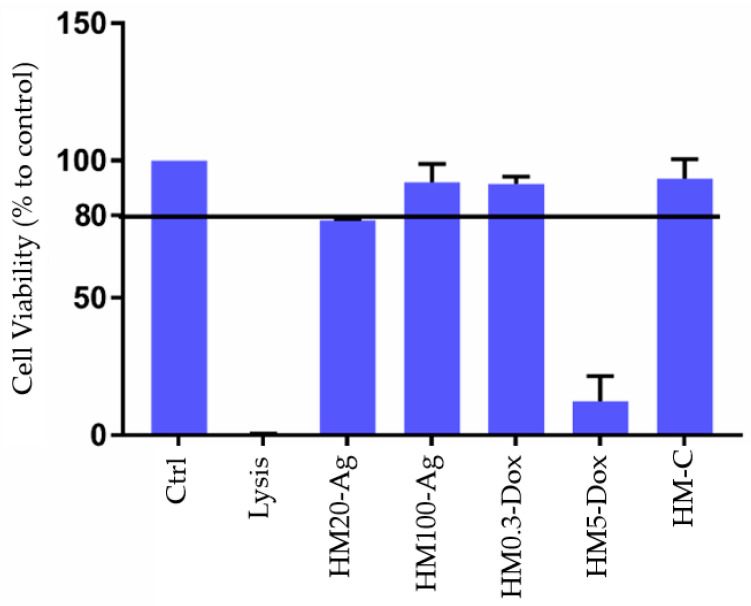
Cell proliferation assay. Bars represent the average of two different experiments, where each experimental condition was tested in triplicate ± S.D. (n = 3, ×2).

The impact on cell proliferation was assessed using the MTS assay, comparing hydrogel-treated normal human fibroblasts with an untreated control group. After 24 h of exposure to the hydrogel samples, the results show diverse effects of the formulations on cell proliferation (Figure 16). According to the literature, materials can be classified as non-cytotoxic if cell viability measures at least 70% after exposure [72,142]. Sample HM5-Dox demonstrated cytotoxicity, exhibiting less than 20% cell viability when compared to the untreated cells. Conversely, samples HM100-Ag, HM0.3-Dox, and HM-C were non-toxic, with over 80% cell viability, whereas sample HM20-Ag viability was slightly less than 80% (78%). These results are similar to the other literature reports where smaller-sized nanoparticles have been shown to have a more toxic effect on different cell lines [143,144,145,146]. The cytotoxic effects of npAg result from mechanisms such as cellular uptake and inflammation, DNA damage, and the release of reactive oxygen species (ROS). These mechanisms are influenced not only by the concentration of nanoparticles but also by their size, shape, surface chemistry, time of exposure, and cell type [147]. Tort et al. found that a coaxial nanofiber mat containing DOXY had good biocompatibility for keratinocytes at 3 and 7 days [79]. Similarly, Cui et al. showed that nanofibers incorporating different concentrations of DOXY had good biocompatibility when tested on mouse fibroblast cells [148]. In our study, sample HM0.3-Dox showed excellent biocompatibility, while the introduction of a higher DOXY concentration (sample HM5-Dox) had a profound impact on cell proliferation.

#### 3.12.3. Cell Migration Assay

**Figure 17:**
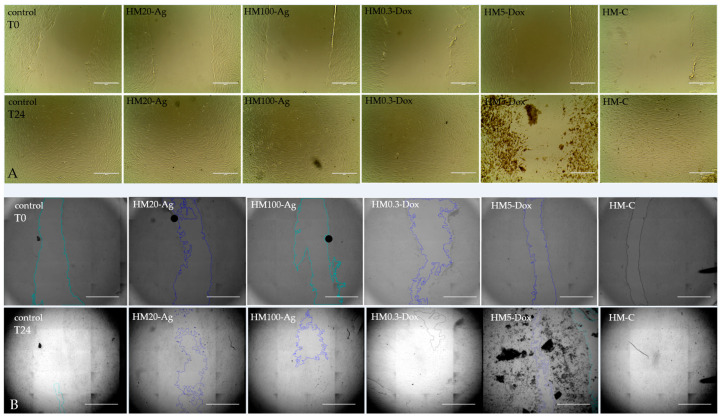
Scratch-wound assay. HS27 human fibroblasts were plated to confluence in 96 wells; a scratch was made in each well (T0, upper images), and triplicates were treated with the hydrogel samples for 24 h (T24, lower images). (**A**) Details of area coverage after 24 h of treatment (Evos XL, phase contrast, 10×, scale bar 400 µm). (**B**) Area coverage analysis was performed on the entire scratch surface for each well, captured by scanning and stitching of 20 fields/well. The composite image was annotated and measured in ImageJ. Images with no markings (control T24 and HM-C 24) had full coverage of the nude area (Evos FL, scale bar 2 mm).

**Figure 18:**
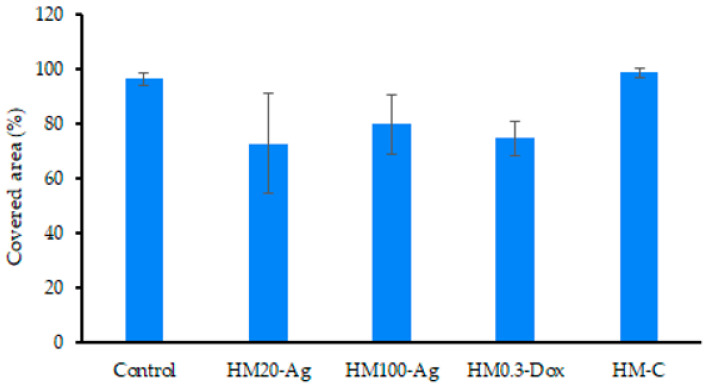
Cell migration assay. The samples were analyzed in triplicate and are presented as mean ± S.D.

Cell migration, as assessed by the scratch-wound assay (Figure 17), showed that sample HM-C performed similarly to the control, and the scratch was closed after 24 h of treatment. The difference in coverage area between control and samples HM20-Ag, HM100-Ag, and HM0.3-Dox did not reach statistical significance (Figure 18). However, the scratch wound assay showed an increase in covered area at 24 h for cells treated with these samples. Because cells treated with the sample HM5-Dox detached rather than proliferate and migrate (as visible in Figure 17), the overall area coverage was negative and was excluded from the analysis. It has been reported that npAg can influence the migration of human fibroblasts, possibly by promoting the formation of intracellular stress fibers [149]. In a previous study, it has been shown that at higher doses, DOXY could inhibit cell proliferation, induce apoptosis, increase cell adhesion, and alter cell morphology in LAM-related cells [150]. Another study revealed that DOXY hindered the process of epithelial-to-mesenchymal transition and migration induced by TGF beta 1 in respiratory epithelial cells [151]. Chang et al. revealed that DOXY inhibited electric field-induced migration of small-cell lung cancer (NSCLC) cells [152]. Our findings align with previous studies highlighting the potential impact of npAg and DOXY on cell behavior, underscoring the need for careful consideration of dosage and application for biomedical applications.

### 3.13. Surface Morphology Analysis

**Figure 19:**
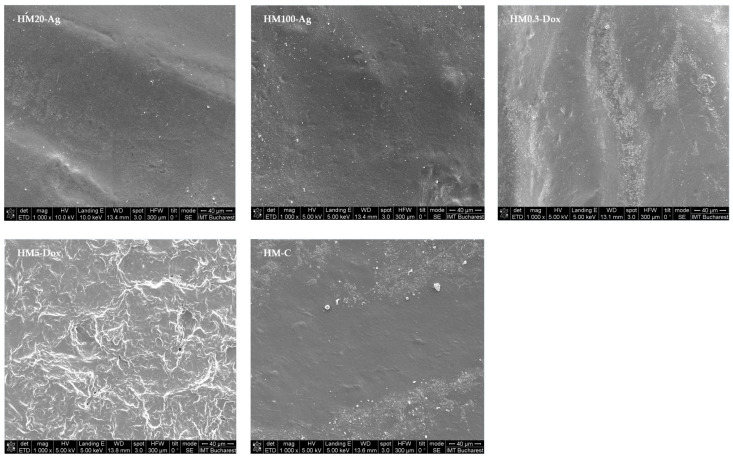
Microstructure analysis of hydrogel membranes using SEM.

The microstructure of the hydrogel was assessed using SEM (Figure 19). Increasing magnification and focus led to the destruction of the hydrogel’s surface in some cases, with the appearance of cracks, so the microscope parameters had to be carefully adjusted [91]. The surface morphology of the hydrogel membranes differs among formulations. The uniform incorporation of different-sized npAg is confirmed for both sample HM20-Ag and sample HM100-Ag; however, some aggregations can be observed. As reported by other authors, npAg appears as a glowing spherical structure within the hydrogel membrane [153,154]. The surface of samples containing npAg is smooth and uniform, and the porosity of the membrane is lost compared to the rest of the samples. The hydrogel membrane appears to be more compact and denser. Wang et al. report that the hydrogel incorporated with npAg maintained the porous structure, although the mean size of the npAg was in the range of 200 nm, and the samples were freeze-dried before analysis [127]. Helmiyati et al. showed by SEM images how npAg with an average size of 20 nm occupies the pores of sodium alginate–polyvinyl alcohol–g–acrylamide hydrogel [99]. The literature reports that the addition of metal nanoparticles can have a nanofilling effect that will modify the hydrogel properties such as mechanical properties, conductivity, antimicrobial action, and other stimuli-responsive properties [87,155,156]. With the addition of DOXY, the porosity of the hydrogel membrane is preserved. The sample HM0.3-Dox showed a similar surface morphology to that of the control, with well-preserved porosity. Sample HM100-Dox exhibited increased surface roughness but also showed several pores, although the distribution was reduced.

## 4. Conclusions

Our extensive investigation of the biohybrid hydrogel membranes underscores their potential as highly effective wound dressings. All samples showed exceptional swelling capabilities except the high-dose DOXY hydrogel. Hydrogels incorporating 100 nm npAg or low-dose DOXY demonstrated acceptable moisture absorption capacities. All samples displayed gel-like properties. However, the incorporation of 100 nm npAg led to a notably stiffer hydrogel compared to the introduction of 20 nm npAg. Similarly, higher doses of DOXY resulted in increased rigidity of the hydrogel when compared to lower doses of DOXY. All samples exhibited a degradation rate of 70% or higher within a 20-h timeframe. High-dose DOXY hydrogel demonstrated superior antioxidant and anti-inflammatory properties when compared to all other samples. Interestingly, the pH value exhibited minimal influence on the cumulative release of DOXY over 24 h. It was observed that the release profile was more significantly affected by the concentration of DOXY, which also exerted a substantial influence on the physicochemical properties of the hydrogel membranes. Furthermore, all DOXY-containing hydrogels exhibited excellent antibacterial properties against the tested microbial strains. Also, hydrogels incorporating 100 nm npAg or low-dose DOXY demonstrated exceptional biocompatibility, yielding good results in terms of cell proliferation and cell migration. These combined characteristics make the biohybrid hydrogel membranes a promising candidate for advanced wound dressing applications. Further comprehensive analyses are warranted to gain a deeper understanding of the properties and behavior exhibited by the hydrogel membranes. This will facilitate a more nuanced exploration of their potential applications, particularly in the context of wound dressings. The absence of X-ray diffraction (XDR) or differential scanning calorimetry (DSC) investigations, which could offer valuable insights into the material’s characteristics, represents a limitation of the current study.

## Figures and Tables

**Table 1:** Formulations of the biohybrid hydrogel membranes.

Sample	HM20-Ag	HM100-Ag	HM0.3-Dox	HM5-Dox	HM-C	HM-Cp
PVA	2.3% (*m*/*v*)	2.3% (*m*/*v*)	2.3% (*m*/*v*)	2.3% (*m*/*v*)	2.3% (*m*/*v*)	2.3% (*m*/*v*)
Alginate	0.6% (*m*/*v*)	0.6% (*m*/*v*)	0.6% (*m*/*v*)	0.6% (*m*/*v*)	0.6% (*m*/*v*)	0.6% (*m*/*v*)
Glycerol	8.57% (*v*/*v*)	8,57% (*v*/*v*)	8.57% (*v*/*v*)	8.57% (*v*/*v*)	8.57% (*v*/*v*)	8.57% (*v*/*v*)
CaCl_2_	0.075% (*m*/*v*)	0.075% (*m*/*v*)	0.075% (*m*/*v*)	0.075% (*m*/*v*)	0.075% (*m*/*v*)	0.075% (*m*/*v*)
Hyaluronic Acid	2 µg/mL	2 µg/mL	2 µg/mL	2 µg/mL	2 µg/mL	-
Collagen	35 µg/mL	35 µg/mL	35 µg/mL	35 µg/mL	35 µg/mL	-
npAg 20 nm	10 µg/mL	-	-	-	-	-
npAg 100 nm	-	10 µg/mL	-	-	-	-
Doxycycline	-	-	0.3 mg/mL	5 mg/mL	-	-

“-” indicates the absence of the reagent.

**Table 2:** Physico-mechanical parameters used during rheological frequency-sweep tests at 37 °C.

Sample	Radius (mm)	Gap (Sample Thickness) (µm)	Amplitude of the Vertical Displacement (µm)
HM-C	9.1	730	0.04
HM20-Ag	9.8	700	0.04
HM100-Ag	9.5	720	0.04
HM0.3-Dox	9.1	780	0.04
HM5-Dox	9.1	770	0.04

**Table 3:** Weight, thickness, and pH variations in obtained samples.

Sample	Weight (g)	Thickness (mm)	pH
HM20-Ag	4.98 ± 0.19	4.5 ± 0.32	7.3 ± 0.02
HM100-Ag	4.92 ± 0.32	4.45 ± 0.47	7.29 ± 0.01
HM0.3-Dox	5.41 ± 0.13	4.78 ± 0.26	7.14 ± 0.02
HM5-Dox	5.47 ± 0.17	4.8 ± 0.18	4.62 ± 0.03
HM-C	4.80 ± 0.15	4.35 ± 0.38	7.34 ± 0.05

Results are presented as mean ± S.D.; n = 3 for weight and pH; n = 5 for thickness.

**Table 4:** Comparative kinetic model of doxycycline release from hydrogel membranes.

Sample	Zero-Order Kinetic		First-Order Kinetic		Higuchi Model		Kosmeyer Peppas Model		
	K	R^2^	K (h^−1^)	R^2^	K (h^−1/2^)	R^2^	K (h^−n^)	n	R^2^
HM0.3-Dox	0.3426	0.3096	−0.0016	0.3188	2.4706	0.5994	0.82783	0.165079	0.943085
HM5-Dox	0.416	0.1411	−0.0019	0.1405	2.4406	0.3776	4.05068	0.051096	0.997314

**Table 5:** Measured inhibition zones (mm) for the tested samples (duplicate).

Bacterial Strain	Inhibition Zone in mm (Duplicate)													
	HM20-Ag		HM100-Ag		HM0.3-Dox		HM5-Dox		HM-C		HM-Cp		DOXI Standard Disk	
*S. epidermidis*	0	0	0	0	17	17	30	30	0	0	0	0	17	16
*S. aureus*	0	0	0	0	19	19	30	30	0	0	0	0	17	17
*E. faecalis*	0	0	0	0	25	25	31	30	0	0	0	0	25	25
*S. pyogenes*	0	0	0	0	25	24	38	35	0	0	0	0	25	25
*P. aeruginosa*	0	0	0	0	18	15	28	26	0	0	0	0	17	18
*A. baumannii*	0	0	0	0	28	30	35	35	0	0	0	0	26	28
*K. pneumoniae*	0	0	0	0	17	15	25	25	0	0	0	0	16	15

## Data Availability

Data are contained within the article.

## Appendix A

**Table A1:** CLSI guidelines (2016). Zones of inhibition dimensions (mm) and interpretations for the tested bacterial strains.

	Susceptible	Intermediate	Resistant
*Streptococcus* spp.	≥23	19–22	≤18
*Staphylococcus* spp.	≥16	13–15	≤12
*Enterococcus* spp.	≥16	13–15	≤12
*Acinetobacter* spp.	≥13	10–12	≤9
*Enterobacterales*	≥14	11–13	≤10
